# Patients’ Perceptions of Quality of Care: A Teamwork Intervention Study in a Surgical Ward

**DOI:** 10.1177/23779608221076814

**Published:** 2022-02-08

**Authors:** Marie Louise Hall-Lord, Randi Ballangrud

**Affiliations:** Department of Health Science, Faculty of Medicine and Health Sciences, 8018 Norwegian University of Science and Technology, Gjøvik, Norway

**Keywords:** continuous quality improvement < business concepts, hospitals, medical/surgical < practice, quantitative research < research

## Abstract

**Introduction:**

Improving teamwork competencies among health care professionals is important for patient safety. Few previous studies have investigated whether a teamwork intervention has an impact on patients’ perceptions of quality of care.

**Objective:**

To investigate patients’ perceptions of quality of care before and after the implementation of a team training program in a surgical ward.

**Methods:**

A quasi-experimental pre- and posttest design was used. The TeamSTEPPS^®^ team training program was implemented in a surgical ward. Three groups of consecutively sampled patients responded to the Quality from the Patient's Perspective (QPP) questionnaire including four dimensions with 25 items. In addition to the QPP, six items were developed for this study. In total, 223 patients responded to the questionnaire. The mean age was 59.6 years, and there were 128 males and 94 females.

**Results:**

The physical-technical condition dimension and four items showed significantly higher scores after six months of intervention. The majority of the patients scored quality of care in the four dimensions as very high at all three time points. Younger patients reported the lowest care quality.

**Conclusion:**

The results in this study indicate that the teamwork intervention had a minimal impact on the patients’ perceptions of quality of care, with only significant differences between baseline and six months of intervention in one dimension and three items. At each data collection time point, the numbers of patients who perceived quality of care as modest decreased slightly. Younger patients were more likely to perceive care quality as modest.

## Introduction

In current specialized and complex health care, patient safety-related incidents may cause harm for patients during hospital stays (WHO, 2021). Collaboration between health care professionals working in interdisciplinary teams has become more important for patient safety (Rosen et al., 2018). A team can be described as two or more individuals who work together to achieve specified and shared goals, have task-specific competencies and specialized work roles, use shared resources, and communicate to coordinate and adapt to change (Brannick & Prince, 1997). The patient should be considered an active member of the health care team, which is important for patient safety (WHO, 2011). The patient is a valuable source of information as an expert in the experience of his or her illness and condition and is the only member of the team who is present at all times during care. Health care professionals should be sensitive to patients’ preferences and provide person-centered care by identifying factors that might influence patients’ preferences for participation in their care (WHO, 2013). This study evaluates the implementation of a team training program in a surgical ward with regard to patients’ perceptions of quality of care.

## Review of Literature

Communication failure between health care professionals (Ammouri et al., 2015) and between health care professionals and patients may threaten patient safety (Burgener, 2017). Poor communication, hierarchical structure of surgical teams and high workload were identified as contributing factors for delayed escalation of patient care after surgical complications, which was associated with higher mortality rates (Johnston et al., 2015).

A lack of effective teamwork jeopardizes the quality and safety of care, therefore improving teamwork through interventions has become a priority (Rosen et al., 2018). Intervention studies based on specific teamwork principles and methods, such as simulation, general team training or team training programs, have been conducted within various health care settings (Buljac-Samardzic et al., 2020). Several of the studies have shown a positive effect of team interventions, such as improved team competence, team performance, safety culture, and clinical outcome (Buljac-Samardzic et al., 2020). Improvements in patients’ perceptions of quality of care were observed after an interprofessional teamwork intervention in an emergency room (Muntlin et al., 2016). Definitions of quality of care vary depending on whose perspective is taken and within which context it is considered. In this study, a theoretical model of quality of care was used as the theoretical foundation. This model, The Quality of Care from the Patients’ Perspective (QPP), views care quality through the patient's eyes. Norms, expectations, experiences and patients’ encounters with the existing care structure constitute patients’ perceptions of quality of care (Wilde et al., 1993).

To improve quality and safety in health care in the US, the Department of Defense and the Agency for Health care Research and Quality (AHRQ) developed the Team Strategies and Tools to Enhance Performance and Patient Safety (TeamSTEPPS^®^) program. This program is built on the “Big Five” framework of effective teamwork (Salas et al., 2005) and includes five key principles: first, team structure; and four important teamwork competencies - communication, leadership, situation monitoring and mutual support. The program focuses on improving team competencies that support team performance, such as training, behavior and culture changes, to improve patient safety (Pettit & Duffy, 2015). Studies implementing the TeamSTEPPS^®^ program have shown intervention effects such as fewer medication errors (Sawyer et al., 2013) and improvements in patient safety culture (Aaberg, Ballangrud, et al., 2019; Thomas & Galla, 2013), health care professionals’ team performance (Mayer et al., 2011; Weaver et al., 2010) and team competency (Harvey et al., 2014; Wong et al., 2016). Studies investigating patient outcomes in relation to implementation of the Team STEPPS^®^ program found a decrease in falls (Godlock et al., 2016), surgical morbidity and mortality, and increased patient satisfaction in terms of willingness to recommend the ward/hospital (Armour Forse et al., 2011).

Most of the TeamSTEPPS^®^ intervention research in hospitals has investigated health care professionals’ satisfaction with the team training, team performance, teamwork attitude, perception of teamwork and efficiency in patient care (Welsch et al., 2018). The research has primarily been conducted in acute hospital settings, such as operating rooms, emergency departments, and intensive care units. Few TeamSTEPPS^®^ intervention studies have been conducted in surgical wards, and to our knowledge, no studies have focused on the impact of an intervention on patients’ perceptions of quality of care. This study is a part of a larger project that mainly evaluated the implementation of the team training program in relation to the staff's perceptions (Ballangrud et al., 2017). The objective of the study was to investigate patients’ perceptions of quality of care before and after the implementation of a team training program in a surgical ward.

## Methods

### Design

The study had a quasi-experimental pre- and posttest design. Three groups of patients who responded to a self-report questionnaire at three time points (T0, T1 and T2) evaluated the quality of care before and after a team training program implemented in a surgical ward. The study was conducted from April 2016 to June 2017.

### Research Questions

We formulated the following research questions:

Are there any differences in patients’ perceptions of quality of care from baseline to 6 months and from baseline to 24 months after the intervention?

Are there any differences between patients’ backgrounds and hospital stay variables in relation to their perceptions of quality of care?

### Sample

The study took place at a 20-bed combined gastrointestinal and urology surgical ward in a hospital in Norway. The reason for selecting this ward was the chief manager's interest in quality improvement. The ward had an average bed occupancy of 87%. The mean patient length of stay was 3.46 days. The number of full-time staff was 13 physicians, 17.25 registered nurses and 4.95 nursing assistants.

A consecutive sample of elective and emergency patients was included on three occasions.

### Inclusion Criteria

The inclusion criteria for the patients were as follows: being 18 years or older, understanding Norwegian, and being in a mental and physical health condition that made it possible to respond to a questionnaire.

### The TeamSTEPPS^®^ Intervention

The intervention was conducted from January 2016 to June 2017. The intervention was organized according to the TeamSTEPPS^®^ model of change, which included three phases (AHRQ, 2012). The implementation was guided by the 8-step Kotter's change model (Kotter, 2012). The implementation of the TeamSTEPPS^®^ intervention has been described in another study (Aaberg et al., 2019b).

*Phase 1) Set the stage and decide what to do - assessment and planning (January 2016 to April 2016)*.

After site assessment, the leaders at the ward considered the ward ready for implementing the TeamSTEPPS^®^ program. A project group including the leaders and the members of the research group was established. Two registered nurses and two physicians from the surgical ward completed a TeamSTEPPS^®^ master training course in the US. Thereafter, the planning of the implementation of the TeamSTEPPS^®^ intervention took place.

*Phase 2) Make it happen - training and implementation (May 2016 to December 2016)*.

All health care professionals at the ward attended one day of six-hour interprofessional team training in a simulation center. The team training, led by the master trained nurses and physicians, included classroom training with lectures, video and role play, and high-fidelity simulation. The team training was conducted on three occasions, and physicians, registered nurses and nursing assistants were trained together. To create a vision and an action plan for the implementation of the TeamSTEPPS^®^ program, a ‘change team’ was established. The ‘change team’ included frontline health care professionals (physicians, registered nurses and nursing assistants), leaders, a former patient and a member of the research group. The action plan was communicated to the health care professionals at the ward. Approximately one tool of the Five TeamSTEPPS^®^ tools were implemented every month (Table 1). Posters were placed at the ward with information on the tools and a request that patients and visitors communicate with a staff member if they noticed something that might threaten patient safety. Patient-targeted actions included dedicated nursing staff being responsible for specific patient rooms, headphones being provided for other patients sharing the same room during the physicians’ bedside rounds and rooms being provided for conversations with patients and their caregivers. Furthermore, the patient was asked to complete a declaration form prior to admission to the hospital to include the patient as part of the team.

**Table 1.** Implementation of TeamSTEPPS Tools and Strategies.

	Tools and strategies	Description
2016		
May	Closed loop *Communication*	Using closed-loop communication to ensure that information conveyed by the sender is understood by the receiver as intended
June	ISBAR *Communication*	A technique for communicating critical information that requires immediate attention and action concerning a patient's condition
August	Briefs *Leadership*	Short session prior to start to share the plan, discuss team formation, assign roles and responsibilities, establish expectations and climate, anticipate outcomes and likely contingencies
September	Huddles *Leadership*	Ad hoc meeting to re-establish situational awareness, reinforce plans already in place, and assess the need to adjust the plan
October	Cross-monitoring *Situation Monitoring*	A harm error reduction strategy that involves; Monitoring actions of other team members; Providing a safety net within the team; Ensuring that mistakes or oversights are caught quickly and easily and Watching each other's back
2017		
January	Debriefs *Leadership*	Informal information exchange session designed to improve team performance and effectiveness through lessons learned and reinforcement of positive behaviors
February	STEP *Situation Monitoring*	Tool to help assess health care situations and involves. Status of Patient; Team Members; Environment and Progress Toward Goal.
March	Two-Challenge Rule *Mutual Support*	Empowers all team members to “stop the line” if they sense or discover an essential safety breach.
May	I-PASS *Communication*	The transfer of information (along with authority and responsibility) during transitions in care across the continuum. It includes an opportunity to ask questions, clarify, and confirm. Examples of transitions in care include shift changes; transfer of responsibility between and among nursing assistants, nurses, nurse practitioners, physician assistants, and physicians; and patient transfers

Note. ISBAR = **I**ntroduction, **S**ituation, **B**ackground, **A**ssessment, **R**ecommendation.

STEP = **S**tatus of the patient, **T**eam members, **E**nvironment, **P**rogress towards the goal.

I-PASS = **I**llness severity, **P**atient summary, **A**ction list, **S**ituation awareness and contingency planning.

TeamSTEPPS 2.0 Pocket Guide.

*Phase 3) Make it stick - sustainment (January 2017 to June 2017)*.

In the third phase, the staff was coached, and the action plan was integrated and evaluated. Another five tools were implemented (Table 1). The health care professionals used the tools in their daily work at the ward. Furthermore, TeamSTEPPS^®^ refresher courses were arranged separately for the nursing staff and the physicians.

### Measurements

The short version of the Quality from the Patient's Perspective (QPP) questionnaire including 25 items was used (Larsson et al., 1998; Wilde Larsson & Larsson, 2002). The questionnaire is based on a theoretical model of quality of care developed from a grounded theory study (Wilde et al., 1993). The model was operationalized into the QPP questionnaire using a conventional factor analytical approach (Wilde et al., 1994). The QPP questionnaire was refined in 1998 using structural equation modeling (Larsson et al., 1998) and further developed into a short version in 2002 with a sample of medical and surgical patients (Wilde Larsson & Larsson, 2002). The QPP questionnaire is divided into four dimensions: medical-technical competence (four items), physical-technical conditions (three items), identity-oriented approach (13 items), and sociocultural atmosphere (five items). The medical-technical competence dimension comprised caregivers’ ability to make correct diagnoses and to provide necessary care and treatment. The physical-technical conditions dimension considered the patient's nutritional intake, a comfortable bed and the availability of medical-technical equipment. The identity-oriented approach dimension included information about care, examinations and treatment, participation in care decisions, and empathic skills of caregivers and their ability to be respectful and show commitment to the patient's needs and wishes. The sociocultural atmosphere dimension included aspects of the patient's next of kin, the atmosphere and privacy in the ward and whether the care was governed by requests and needs of the patient rather than routines. The response options are answered on a 4-point Likert scale, ranging from 1 = ‘Do not agree at all’ to 4 = ‘Completely agree’. Each item has a ‘Not applicable’ response alternative. Each dimension is calculated by adding the item scores to a total score and dividing the score by the number of items in the dimension. The psychometric properties of the questionnaire have been found to be satisfactory (Larsson et al., 1998; Wilde Larsson & Larsson, 2002). The reliability of the QPP questionnaire has been measured in different patient populations, and the questionnaire is widely used in both research and clinical improvement initiatives. The Cronbach alpha values in this study were as follows: medical-technical competence dimension = .77, physical-technical conditions dimension = .56, identity-oriented approach dimension = .92, and sociocultural atmosphere dimension = .79.

The QPP dimensions and item scores ranging between 3.30 and 4.0 are considered to indicate very high quality of care, scores between 3.30 and 3.00 are considered to indicate high care quality, and scores lower than 3.00 are considered to indicate modest quality of care (Wilde Larsson, 1999).

In addition to the QPP, we developed six items specifically for this study, inspired by a questionnaire used in another teamwork study (Auerbach et al., 2012) and the TeamSTEPPS^®^ Teamwork Perceptions Questionnaire (T-TPQ) (Keebler et al., 2014). These six items are not a part of QPP, but the items were formulated in the same way as the QPP items and had the same response options.

In addition, five background questions and three questions about the patients’ hospital stay were included.

### Data Collection

The data collection was performed on three occasions: at baseline (T0), six months after the team training (T1) and after 12 months of intervention (T2). On each occasion, the data collection lasted for six weeks. The day before discharge or the day of discharge, the responsible registered nurse assessed whether the patient was in a physical and mental condition that they could respond to a questionnaire. The patient received oral and written information about the study, and after consenting to take part in the study, the patient completed the questionnaire anonymously and put the questionnaire in a sealed envelope. The questionnaires were kept locked in the ward until one of the researchers collected them.

### Statistical Analysis

To determine the number of patients, a power analysis was performed. To detect a mean difference of 0.4 in the score for the item ‘participate in the decisions applied to my care’ (the primary endpoint), sample sizes of 65 (baseline), 80 (after 6 months) and 80 (after 12 months) (alpha <.05, power 0.80, standard deviation of 0.9) were needed to find a significant difference between groups. The data were analyzed using SPSS version 26.0 (IBM Corporation, Armonk, NY, USA). Descriptive statistics, including the frequency, percentage, mean, and standard deviation, were calculated to describe the sample and the patients’ perceptions of quality of care. The chi-square test was used to compare background and hospital stay variables of the three samples. The Mann–Whitney U test was conducted to compare differences in the patients’ perceptions of quality of care between T0 and T1 and between T0 and T2. The patients were divided into three groups according to the QPP dimension scores (very high, high, and modest care quality). Subgroup analysis with the chi-square test was performed to explore differences in the background and hospital stay variables. A p value of *p* < .05 was considered statistically significant. The checklist Transparent Reporting of Evaluations with Nonrandomized Designs (TREND) was used (Des Jarlais et al., 2004).

## Results

### Sample Characteristics

In total, 223 out of 306 eligible patients responded to the questionnaire over the three data collection time points. The mean age of the total sample was 59.6 (*SD* = 18.2), and there were 128 males and 94 females.

Fifty-two patients responded at T0, 89 patients responded at T1, and 82 patients responded at T2. Table 2 describes the background and hospital stay variables of the responding patients. There were no significant differences in these variables between the patients who responded at the three time points. In total, 83 patients did not consent to participate in the study (T0 = 6 patients, T1 = 52 patients and T2 = 25 patients). At each data collection time point, there were no significant differences in age or sex between the participating patients and the patients who did not consent to participate.

**Table 2.** Background and Hospital Stay Data of the Patients at Three Measures.

Variables	T0	T1	T2
	*N* = 52	*N* = 89	*N* = 82
Age md(range), n(%)	66.5 (19–87)	63 (19–90)	61 (18–87)
19–49	9 (17.3)	23 (25.8)	21 (26.3)
50–74	33 (63.5)	46 (51.7)	42 (52.5)
75–90	10 (19.2)	20 (22.5)	17 (21.3)
Missing	0	0	2
Sex n(%)			
Male	37 (71.2)	46 (51.7)	45 (55.6)
Female	15 (28.8)	43 (48.3)	36 (44.4)
Missing	0	0	1
Education level n(%)			
Compulsory school	20 (38.5)	32 (36.0)	15 (19.0)
Upper secondary school	22 (42.3)	34 (38.2)	46 (58.2)
University/University college	10 (19.2)	23 (25.8)	18 (22.8)
Missing	0	0	3
Living condition n(%)			
Living with other	40 (76.9)	54 (60.7)	57 (71.3)
Living alone	12 (23.1)	35 (39.3)	23 (28.7)
Missing	0	0	2
Occupation n(%)			
Employee	17 (32.7)	35 (39.8)	31 (39.2)
Retirement pension	26 (50.0)	43 (48.9)	38 (48.1)
Student	2 (3.8)	1 (1.1)	7 (8.9)
Other	7 (13.5)	9 (10.2)	3 (3.8)
Missing	0	1	3
Admission type n(%)			
Emergency	21 (41.2)	43 (48.9)	33 (41.8)
Scheduled	30 (58.8)	45 (51.1)	46 (58.2)
Missing	1	1	3
Length of hospital stay n(%)			
0–1 days	16 (31.4)	22 (24.7)	22 (27.5)
2–3 days	16 (31.4)	31 (34.8)	34 (42.5)
4–9 days	14 (27.5)	29 (32.6)	22 (27.5)
10 or more days	5 (9.8)	7 (7.9)	2 (2.5)
Missing	1	0	2
Previous admittance to hospital within last month n(%)			
Yes	40 (78.4)	58 (65.9)	57 (72.2)
No	11 (21.6)	30 (34.1)	22 (27.8)
Missing	1	1	3

### Research Question Results

At baseline (T0), the highest QPP scores were found for the medical-technical competence and sociocultural atmosphere dimensions (Table 3). The following three items had the highest scores: ‘nurses were respectful’, ‘pleasant atmosphere in the ward’, and ‘staff worked well together in teams’. The lowest score was found for the added item, ‘participate in decisions applied to medical treatment’.

**Table 3.** Comparisons of Patients’ Perceptions of Quality of Care Between T0 and T1.

Dimension Item^1^	T0 *N* = 52	T1 *N* = 89	Mann Whitney U test	
	M/SD	M/SD	Z	*p*
**Medical-technical competence**	3.55/.63	3.66/.51	−.794	.427
Best possible medical treatment	3.71/.70	3.78/.50	−.238	.812
Effective pain relief	3.51/.85	3.79/.52	−2.024	.**043**
Examinations and treatment within acceptable waiting time	3.61/.72	3.59/.82	−.314	.753
Best possible help to take care of my personal hygiene	3.31/.85	3.46/.69	−.728	.466
**Physical-technical condition**	3.40/.54	3.62/.51	−2.493	.**013**
Food and drink that I like	3.60/.71	3.73/.57	−1.078	.281
Comfortable bed	3.10/.98	3.44/.78	−1.955	.051
Access to necessary apparatus and equipment	3.50/.67	3.70/.55	−1.786	.074
**Identity-oriented approach**	3.50/.56	3.60/.53	−1.449	.147
Information about my heath condition	3.62/.60	3.67/.57	−.437	.662
Information on examinations and treatments	3.58/.78	3.63/.72	−.402	.688
Information on the results of examinations and treatments	3.42/.87	3.59/.67	−.888	.375
Information on possible risks and side effects with planned care and treatment	3.23/.91	3.57/.68	−2.050	.**040**
Information on effects and use of medicine	3.41/.73	3.51/.76	−.990	.322
Information on how to take care of myself	3.12/.92	3.19/.90	−.426	.670
Doctors understood my situation	3.44/.79	3.55/.76	−1.123	.262
Doctors were respectful	3.68/.65	3.76/.59	−.865	.387
Doctors showed commitment	3.50/.75	3.68/.62	−1.484	.138
Nurses understood my situation	3.60/.63	3.60/.63	−.061	.952
Nurses were respectful	3.85/.42	3.83/.46	−.146	.884
Nurses showed commitment	3.75/.52	3.71/.64	−.003	.997
Participate in the decisions applied to my care	3.20/.93	3.36/.84	−.868	.385
**Sociocultural atmosphere**	3.58/.50	3.59/.55	−.386	.700
Next of kin treated well	3.75/.60	3.82/.46	−.299	.765
Talked to the doctors in private	3.64/.64	3.45/.82	−.881	.378
Talked to the nurses in private	3.32/.84	3.43/.83	−.668	.504
Care determined by my own requests and needs	3.24/.80	3.37/.78	−.947	.344
Pleasant atmosphere in the ward	3.77/.48	3.76/.53	−.126	.899
**Added items**				
Participate in decisions applied to medical treatment	2.93/.96	3.34/.81	−2-301	.**021**
Staff provided relevant information as soon as possible	3.50/.68	3.61/.63	-1.058	.290
When communicating, the staff allowed enough time for questions	3.64/.60	3.60/.66	−.246	.806
Received unambiguous consistent information from staff	3.50/.71	3.48/.71	−.147	.883
Staff worked well together in teams	3.77/.43	3.73/.50	−.223	.823
Staff prevented mistakes and unwanted events from affecting my care	3.67/.63	3.75/.54	−.695	.487

Note. ^1^Scale could range from 1 (do not agree at all) to 4 (completely agree).

Bold values significant at *p* < .05.

After six months of intervention (T1), the highest QPP scores were found for the medical-technical competence dimension, and the two items ‘nurses were respectful’ and ‘next of kin treated well’. The item ‘information on how to take care of myself’ had the lowest scores.

Significantly higher scores were found between patients at T1 than T0 on the physical-technical condition dimension and on the items ‘effective pain relief’, ‘information on possible risks and side effects with planned care and treatment’, and ‘participate in the decisions applied to medical treatment’.

After 12 months of intervention (T2), the highest QPP scores were found for the medical-technical competence dimension (*M* = 3.69) and the items ‘nurses were respectful’ (*M* = 3.90), ‘next of kin treated well’ (*M* = 3.90), and ‘staff worked well together in teams’ (*M* = 3.87). The items ‘information on how to take care of myself’ (*M* = 3.28) and ‘participate in decisions applied to medical treatment’ (*M* = 3.21) scored the lowest.

No significant differences between T0 and T2 were revealed.

Table 4 displays the numbers and percentages of patients according to the three groups, i.e., those who reported very high, high and modest care quality for the four dimensions. Most of the patients scored the care quality in all dimensions as very high at the three time points.

**Table 4.** Patients with Very High, High and Modest Perceived Care Quality at Three Measures.

	T0 *N* = 52			T1 *N* = 89			T2 *N* = 82		
Dimensions	Very high n/%	High n/%	Modest n/%	Very high n/%	High n/%	Modest n/%	Very high n/%	High n/%	Modest n/%
Medical-technical competence	38/73	8/15	6/12	68/82	7/8	8/10	61/78	14/18	3/4
Physical-technical conditions	32/65.3	10/20.4	7/14.3	69/81	7/8	9/11	59/78	11/14	6/8
Identity-oriented approach	38/73	4/8	10/19	69/78.4	10/11.4	9/10.2	64/80	8/10	8/10
Sociocultural atmosphere	38/76	6/12	6/12	67/79	10/12	8/9	57/75	11/14.5	8/10.5

Subgroup analysis explored possible differences in the background and hospital stay variables between the three care quality groups regarding each dimension at the three time points.

At T0, in the medical-technical competence dimension, the 19–49 years age group (33.3%) was more likely to score the care quality as modest than the other age groups (50–74 years; 9.1% and 75–90 years; 0%) (*p value* = .01). The same pattern was shown in the identity-oriented approach dimension. The youngest age group, i.e., 19–49 years (55.6%), was more likely to score care quality as modest than the older age groups, i.e., those aged 75–90 years (20%) and 50–74 years (9.1%), (*p value* = .03). Furthermore, in the physical-technical conditions dimension, more patients (90%) who had previous admittance to the hospital within the last month scored care quality as modest than patients who did not have previous admittance to the hospital (5.3%) (*p value* = .02).

At T1, more patients in the 19–49 years age group (30%) scored care quality as modest for the identity-oriented approach dimension than the patients in the 50–74 years (11.1%) and 75–90 years (5.0%) age groups (*p value* = .01).

At T2, a higher proportion of patients in the 19–49 years age group (25%) scored care quality as modest for the physical-technical conditions dimension than the patients in the other age groups (50–74 years; 2.5% and 75–90 years; 0%) (*p value* = .01).

## Discussion

The aim of the study was to investigate patients’ perceptions of quality of care before and after implementation of a team training program in a surgical ward. The participating patients’ QPP scores increased significantly only from T0 to T1 for one dimension (physical-technical conditions), for one item in the medical-technical competence dimension, for one item in the identity-oriented approach dimension, and for one added item. After a teamwork intervention in an emergency room, the results showed significantly increased care quality after the first intervention phase and at follow-up after 1.5 years in relation to seven items in three dimensions (medical-technical competence, identity-oriented approach, and sociocultural atmosphere) (Muntlin et al., 2006). Hence, the authors found no improved quality of care in the physical-technical conditions dimension.

In the present study, the mean score for the item “effective pain relief increased significantly” increased from T0 to T1. In another intervention study in a surgical ward, this item had a high score in both the intervention and control groups (Jangland et al., 2012). The high scores of care quality regarding pain relief may more likely be due to enhanced postoperative pain management than to the intervention having an impact. Previous research has shown that less than half of patients report adequate postoperative pain relief. Therefore, guidelines with several strategies and recommendations for reducing and managing postoperative pain have been developed by a multidisciplinary expert panel (Chou & Fasano, 2016).

The score for the item ‘information on possible risks and side effects with planned care and treatment’ improved significantly between T0 and T1. A similar pattern was found for the added item ‘participate in decisions applied to medical treatment’. This item had modest care quality at T0 but reached a very high care quality at T1. The positive changes may be a result of the actions taken to encourage the patient's participation as part of the team. After a teamwork and communication intervention, an increased score for the item ‘participation in decisions applied to medical treatment’ from phase 1 to phase 2 was observed (Auerbach et al., 2012). To allow patients to participate in decisions regarding medical treatment, it is important to inform them about the possible risks and side effects. In a study of collaboration between physicians and nurses, the item ‘patient participation in decision-making’ had the lowest score in medical/surgical wards (Aaberg et al., 2019a). On the other hand, another study found that the staff and especially the physicians in surgical care considered information to be the most important for patient involvement (Andersson et al., 2021). Lack of time and prioritization of other tasks might be barriers to patient involvement. Patients can contribute to staff decisions since they have important information about their health (Zavala et al., 2018). Patients who scored high for participation in decisions in medical/surgical settings were less likely to have at least one adverse event during their hospital stay (Weingardt et al., 2011). To enhance patient safety, it is important to encourage patient participation and involve the patient as part of the team.

In the present study, most patients scored care quality as very high or high in all four dimensions. The proportion of patients who scored care quality as modest in three dimensions except for the physical-technical conditions decreased slightly between T0 and the other two measures. The youngest age group was most likely to score care quality as modest. It is well established in the literature that older patients score quality of care higher than younger patients (Grondahl et al., 2011; Muntlin et al., 2008).

The tools included in the TeamSTEPPS^®^ were implemented gradually during the intervention, and the teamwork competencies improved among the staff and thus improved patient safety in the surgical ward (Aaberg et al., 2021). The intervention affected the behavior and communication of the staff in their daily clinical practice at a surgical ward by allowing them to obtain a greater awareness and knowledge of their teamwork skills. Improved teamwork competencies were described as a more systematic interprofessional information exchange and a shared understanding of accountability and transparency, among others (Ballangrud et al., 2020).

## Strengths and Limitations

The strengths of the study were that it was the first study to investigate the implementation of the TeamSTEPPS^®^ program in relation to patients’ perceptions of quality of care and that the intervention was carried out systematically and in accordance with the TeamSTEPPS^®^ manual. The main limitation concerns the absence of control groups and the use of random assignment with controls would have been preferable. For practical reasons, a quasi-experimental pre- and posttest design may be useful in clinical settings, but caution must be taken in generalizing the results (Polit & Beck, 2017). We considered control groups in another surgical ward, but unfortunately, it was difficult to collect data. We are aware that the design is not the best, but we were interested in possible differences after the intervention in the specified place. We do not expect the exact effect of the intervention, but it can be seen as a first step in the process. Another weakness may be that the questionnaire was not sensitive enough to measure differences. The desired sample size of at least 65 patients at T0, which, according to the power analysis, was needed, to find a significant difference between the groups, was not achieved. At T0, there may be a lower number of patients because the staff did not inform all patients about the study but instead prioritized other tasks in the ward. The staff at the ward decided which patients should be informed and invited to participate in the study. The most seriously ill patients were probably not invited. Only patients who were in a mental and physical condition that allowed them to answer the questionnaire were included. However, the staff may have had varying views of patients’ conditions. Another limitation might be that the patients felt obliged to respond positively when they completed the questionnaire in the ward. However, the patients responded to the questionnaire anonymously, and their experiences of their hospital stay were still recent and not biased by time. Only some weeks after discharge, patients tended to regard care quality more positively, even though their perceptions also depended on their health and recovery (Crow et al., 2002). Another limitation may be how well the QPP and the added items correspond to the content of the team training intervention, even though the QPP is well known and often used to measure care quality.

## Implications for Practice

The TeamSTEPPS^®^ intervention focused on team training and implementation of tools and strategies to improve patient safety during the study period. While we found a small impact on the patients’ perceived care quality in this study, the results showed that the TeamSTEPPS^®^ program influenced quality and safety by encouraging effective teamwork and communication skills. Patient safety can be improved when patients are informed and participate in their care and treatment and are included as part of the team.

## Conclusions

This study concluded that a teamwork intervention had a small impact on patients’ perceptions of quality of care. There were only significant differences between baseline and six months of intervention in the physical-technical conditions dimension and three items: ‘effective pain relief’, ‘information on possible risks and side effects with planned care and treatment’, and ‘participate in the decisions applied to medical treatment’. However, the number of patients who perceived quality of care as modest decreased slightly at each data collection time point. Most patients perceived quality of care as very high or high. Patients in the youngest age group were more likely to perceive care quality as modest. It can be a challenge to show the importance of an intervention in terms of perceptions of quality of care because different groups of patients participated at the three time points. To measure the impact of a TeamSTEPPS^®^ intervention in relation to patients’ perceptions of care quality, further studies are required, and it is necessary to develop relevant interventions. We also recommend using a control group for future studies.
